# Posttraumatic growth and illness perception in survivors of adolescent and young adult cancer

**DOI:** 10.1007/s12672-023-00810-3

**Published:** 2023-10-30

**Authors:** Céline Bolliger, Pauline Holmer, Silvia Dehler, Katharina Roser, Gisela Michel

**Affiliations:** 1 Faculty of Health Sciences and Medicine, University of Lucerne, Lucerne, Switzerland; 2 Cancer Registry Zurich and Zug, Institute of Surgical Pathology, University Hospital Zurich, Zurich, Switzerland; 3 Epidemiology, Biostatistics and Prevention Institute, University of Zurich, Zurich, Switzerland; 4 Office of Public Health, Vaduz, Principality of Liechtenstein

**Keywords:** Adolescent and young adult, Cancer, Survivor, Posttraumatic growth, Benefit finding, Illness perception, Oncology, Survey and questionnaire, Cancer registry

## Abstract

**Background:**

Adolescents and young adults (AYAs) are diagnosed with cancer during a challenging period of life. We aimed to (1) describe positive changes (posttraumatic growth; PTG) and illness perception, and (2) determine associations between PTG and illness perception, sociodemographic, and cancer-related characteristics in Swiss AYA cancer survivors.

**Methods:**

We conducted a population-based survey among AYA cancer survivors diagnosed 1990–2005 at age 16–25 years, who had survived ≥ 5 years. We used the Posttraumatic Growth Inventory (PTGI) and the Brief Illness Perception Questionnaire (BIPQ). Data were analyzed using descriptive statistics and linear regressions.

**Results:**

Among 389 contacted survivors, 160 responded (61.3% male; mean age = 34 years, SD = 5.8). The mean PTG sum score was 54.63 (SD = 20.24; range: 8–101). Survivors reported high PTG especially in the domains Appreciation of life (mean = 3.23; 95% confidence interval, 3.05–3.40), Personal strength (2.94; 2.77–3.12), and Relating to others (2.57; 2.40–2.74). Neither sociodemographic nor cancer-related characteristics were associated with PTG. Survivors who perceived follow-up care as helpful (p < 0.001) and those with high concerns about the consequences of the illness (p < 0.001) reported higher PTG.

**Conclusions:**

Finding ways to promote PTG and to identify and address maladaptive illness perceptions may help survivors transform their experience into something meaningful for their future life.

## Introduction

Struggling with difficult life circumstances, such as getting diagnosed with cancer, can result in positive changes such as posttraumatic growth (PTG) later in life [1]. PTG describes a phenomenon in which individuals experience positive changes that surpass their pre-existing state after facing and overcoming a burdening experience [2]. Individuals who experience PTG may nonetheless experience negative consequences related to the traumatic event [3]. PTG can be experienced as increased appreciation of life, deeper relationships, personal strength, recognition of new possibilities, and spiritual development [2]. There is a growing body of literature suggesting the existence of perceived positive outcomes in various populations of cancer survivors [4–6]. Several factors such as demographic, cancer-related, and psychosocial characteristics were found to be associated with PTG [7–10]. Another factor possibly influencing survivorship might be illness perception. Illness perceptions are personal ideas and mental representations of what people know about their disease, its symptoms, potential causes, timeline, disease progression, and expectations about the consequences [11–13]. Most studies in patients affected by cancer at a younger age so far focused on perceptions during acute illness rather than on the period following the illness when individuals have to cope with possible late consequences [14–16]. There is evidence that the processing of trauma-related cognitions may influence the formation of PTG [2].

Late outcomes and psychosocial needs after the cure of cancer in adolescents and young adults (AYAs, 15–25 years) are unique in this specific age group [17, 18]. Experiencing cancer at this young age is an additional burden in a challenging phase of life when young people are confronted with various developmental tasks [19–24]. Currently, there is limited research focusing on positive experiences that may arise in AYA cancer survivors, and so far, illness perception has not been investigated as a potential factor influencing the development of PTG in AYA cancer survivors [25–27].

We therefore aimed to 1) describe PTG and illness perception in Swiss AYA cancer survivors and 2) determine associations between PTG and illness perception, sociodemographic, and cancer-related characteristics.

## Materials and methods

### Sample and procedure

We identified AYA cancer survivors through the population-based Cancer Registry Zürich and Zug, Switzerland. We included AYA cancer survivors who were between 16 and 25 years of age and resident in the Canton of Zurich at their initial cancer diagnosis, had a cancer diagnosis of leukemia, germ cell tumor, lymphoma, central nervous system (CNS) tumor, neuroblastoma, renal, hepatic and bone tumor, or soft tissue sarcoma between 1990 and 2005, and survived at least 5 years (i.e., at least 5 years after diagnosis). The cancer diagnoses and age range were restricted to enable comparison with a cohort of Swiss childhood cancer survivors [28, 29]. Addresses of eligible survivors were obtained from the cancer registry and updated by contacting the previous community of residence, if necessary. We sent the participants a cover letter, the study information, a consent form, the questionnaire, and a pre-paid return envelope. After 4 weeks, we sent a reminder letter and the same survey to those who had not responded. We conducted the survey between August 2010 and January 2012. All procedures performed in this study were in accordance with the ethical standards of the responsible research committee and with the 1964 Helsinki Declaration and its later amendments or comparable ethical standards. The Cantonal Ethics Committee of Zurich approved the study (EK: 2010–0228/2).

### Measurements

The questionnaire included self-reported information about PTG, illness perception, and sociodemographic characteristics. We also assessed information on follow-up care and psychological distress [30–33]. Cancer-related information was obtained from the registry.

#### PTG

PTG was assessed using the German version of the Posttraumatic Growth Inventory (PTGI) [1]. The PTGI consists of 21 items, is well-validated and comprises five domains: *Relating to others* (7 items), *New possibilities* (5 items), *Personal strength* (4 items), *Spiritual change* (2 items) and *Appreciation of life* (3 items) [1]. Each item is rated using a 6-point Likert scale (0 = not at all to 5 = extremely). We calculated the mean for each of the five domains (range: 0–5). In addition, we calculated the PTG sum score as the sum of all item scores (range: 0–105), with higher scores indicating greater levels of PTG. Finally, we calculated the PTG mean score being the mean of all item scores (range: 0–5). Cronbach’s alpha assessing internal consistency was substancial for our sample for the five domains and overall PTG: Relating to others α = 0.88, New possibilities α = 0.86, Personal strength α = 0.77, Spiritual change α = 0.93, Appreciation of life α = 0.83, and overall PTG α = 0.94, as also shown in Tedeschi and Calhoun [1].

#### Illness perception

A modified version of the Brief Illness Perception Questionnaire (BIPQ) [34], adapted to the situation of cancer survivorship, was used to assess illness perception [35]. It consists of 8 items assessing how the previous cancer and possible late effects affect survivors. Survivors could express their accordance on an 11-point Likert scale. Five items assessed cognitive illness perception: *Consequences* (how much do the consequences of your illness affect your life?), *Timeline* (how long do you think the consequences of your past illness will continue?), *Personal control* (how much control do you feel you have over the consequences of your illness?), *Treatment control* (how much do you think follow-up care can help with the late effects of your illness?), and *Identity* (How much do you still experience symptoms from your past illness?). Two items assessed emotional illness perception: *Concerns* (how concerned are you about the consequences of your illness?) and *Emotions* (how well do you feel you understand your illness consequences?). One item assessed *Illness comprehensibility* (how well do you understand your past illness?). The BIPQ has a good test–retest reliability [34].

#### Sociodemographic characteristics

We assessed survivors’ age (assessed continuously and categorized into 20–39, 30–39 and ≥ 40 years), sex (female, male), educational achievement (compulsory schooling, vocational training, upper secondary education, university degree [29]), being in a partnership (yes, no), and migration background (survivors were classified as having a migration background if they were not Swiss citizen since birth or were not born in Switzerland) in the questionnaire. We asked survivors whether they had any late effects, a cancer relapse, or second malignancies (yes, no) and if they were afraid that late effects are detected when attending follow-up care (7-point Likert scale, 1 = completely agree to 7 = disagree).

#### Cancer-related information from the registry

We obtained information on diagnosis (classified according to the International Classification of Childhood Cancer, Third Edition—ICCC-3 [36]: leukemia, lymphoma, CNS tumor, other tumors), age at diagnosis (continuous), treatment (hierarchically coded as surgery only, chemotherapy (may have had surgery), radiotherapy (may have had surgery and/or chemotherapy)), and time since diagnosis (continuous) from the cancer registry.

### Statistical analysis

We performed all statistical analyses using Stata 17 (StataCorp, College Station, TX). First, descriptive statistics and frequencies were calculated for demographic and cancer-related characteristics from the cancer registry to compare participants with non-participants. For this comparison, we calculated chi-square tests for categorical variables and independent t-tests for continuous variables. Second, we used descriptive statistics to describe PTG and illness perception. We replaced missing PTG values with the mean of the respective domain if at least half of the items within that domain were available (*Relating to others* n = 3, 2%; *Personal strength* n = 3, 2%).We did not impute missing values for illness perception since each item represents a different underlying concept (missing values: *Consequences* n = 6, *Timeline* n = 5, *Personal control* n = 5, *Treatment control* n = 2, *Identity* n = 2, *Concerns* n = 2). In addition, we calculated the proportion of participants endorsing single items at a low (0–1), middle (2–3) or high level (4–5) for PTG. Finally, we ran univariable linear regression analyses to investigate the associations of *PTG sum score* with illness perception, sociodemographic, and cancer-related variables. We included a priori sex [10] and all variables significant at a p < 0.1 level in the multivariable regression model.

## Results

### Sample characteristics

Among 469 cancer survivors eligible for the study, we were able to contact 389 (82.9%) with a current address. Of those, 160 (41.1% of contacted survivors) returned the questionnaire and were included in the analysis (Table 1). The mean age of participants at study was 34.0 years (standard deviation (SD) = 5.8, range: 20.9–46.5 years), and 98 (61.3%) of them were males. The mean age at diagnosis was 21.6 years (SD = 2.9). The most common diagnosis among the participants was lymphoma (n = 60, 37.5%), followed by germ cells tumors (n = 46, 28.7%), CNS tumors (n = 15, 9.4%), and soft tissue sarcomas (n = 15, 9.4%). Regarding treatment, 57 of participating survivors were treated with surgery only (n = 35.6%), 41 (25.6%) with radiotherapy, and 36 (22.5%) with chemotherapy. More than a quarter of AYA cancer survivors (n = 45, 28.1%) reported suffering from late effects. Participants and non-participants were similar regarding all characteristics available from the cancer registry (Table 1).

**Table 1:** Characteristics of the study population, comparing survivor participants and non-participants

AYA cancer survivors (n = 469)					
	Participants (n = 160)		Non-participants (n = 309)		p value^d^
Sociodemographic characteristics	n	%	n	%	
Sex^a^					0.110
Male	98	61.3	210	68.0	
Female	62	38.7	96	31.0	
Age at study					0.596
20–29 years	43	26.9	74	24.0	
30–39 years	85	53.1	180	58.2	
≥ 40 years	32	20.0	55	17.8	
Migration background					n.a
No	125	78.1	–	–	
Yes	35	21.9	–	–	
Educational achievement^a^					n.a
Compulsory schooling	13	8.1	–	–	
Vocational training	74	46.3	–	–	
Upper secondary education	53	33.1	–	–	
University education	19	11.9	–	–	
Employment status^a^					n.a
Employed	145	90.6	–	–	
Unemployed	14	8.8	–	–	
Partnership					n.a
Yes	123	76.9	–	–	
No	37	23.1	–	–	

Cancer-related characteristics	n	%	n	%	
Diagnosis (ICCC-3)					0.356
Leukemia	13	8.1	28	9.1	
Lymphoma	60	37.5	91	29.5	
CNS tumor	15	9.4	36	11.6	
Neuroblastoma^b^	2	1.3	2	0.6	
Renal tumor^b^	3	1.9	1	0.3	
Hepatic tumor^b^	0	0.0	2	0.6	
Bone tumor^b^	6	3.7	15	4.9	
Soft tissue sarcoma^b^	15	9.4	17	5.5	
Germ cell tumor^b^	46	28.7	117	37.9	
Treatment^a,c^					0.482
Surgery	57	35.6	109	35.3	
Chemotherapy	36	22.5	75	24.3	
Radiotherapy	41	25.6	60	19.4	
Age at diagnosis					0.976
16–21 years	70	43.8	122	39.5	
> 21–25 years	90	56.2	187	60.5	
Time since diagnosis					0.976
5–10 years	59	36.9	111	36.0	
11–15 years	51	31.9	99	32.0	
≥ 16 years	50	31.2	99	32.0	
Late effects^a^					n.a
No	111	69.4	–	–	
Yes	45	28.1	–	–	
Relapse					n.a
No	136	85.0	–	–	
Yes	24	15.0	–	–	
Second cancer					n.a
No	148	92.5	–	–	
Yes	12	17.5	–	–	

	Mean	SD	Mean	SD	
Age at study	34.0	5.8	34.2	5.6	0.754
Age at diagnosis	21.6	2.9	21.7	2.9	0.706
Time since diagnosis	12.4	4.8	12.5	4.8	0.884

*AYA adolescent and young adult, *CNS* central nervous system, *ICCC-3* International Classification of Childhood Cancer—Third Edition, *n* number, *n.a.* not available, *SD* standard deviation

^a^Missing values; percentages are based on the total number of participants/non-participants

^b^Neuroblastoma, renal tumor, hepatic tumor, bone tumor, soft tissue sarcoma, and germ cell tumor were combined in a category called “other tumors” for the analyses

^c^Hierarchically coded: chemotherapy may include surgery, radiotherapy may include surgery and/or chemotherapy

^d^p-value calculated from t-test statistics (continuous variables) and chi-square test statistics (categorial variables) comparing participants and non-participants

### PTG

**Fig. 1:**
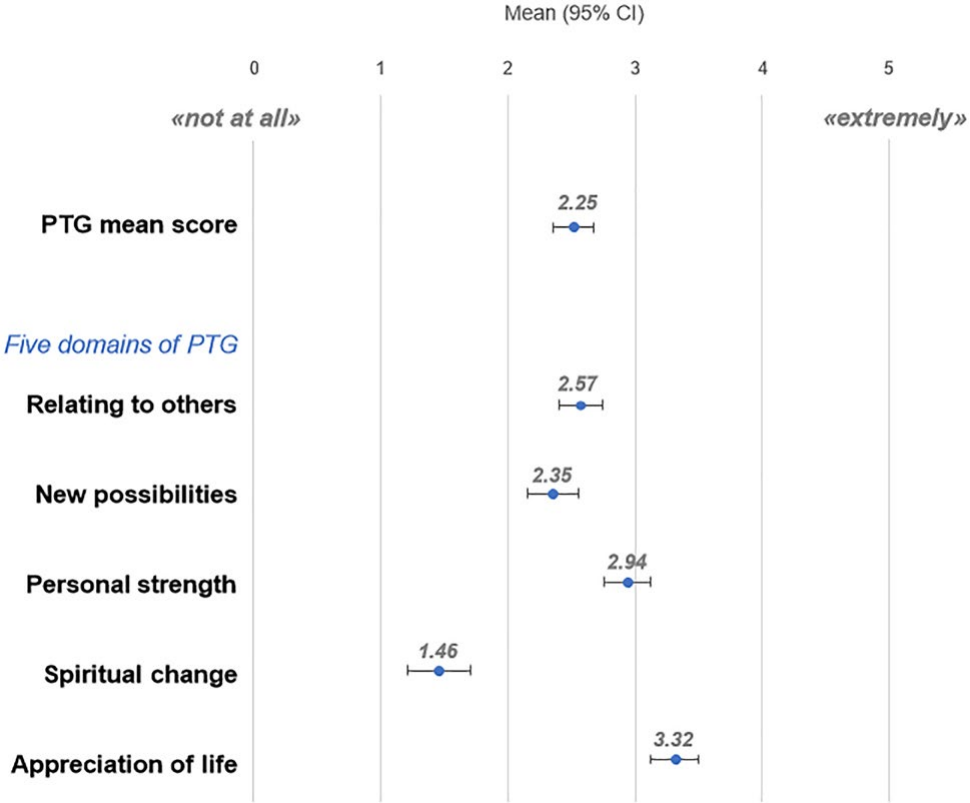
Means and 95% confidence intervals (CI) of the posttraumatic growth (PTG) mean score and the PTG domains measured by the Posttraumatic Growth Inventory (PTGI)

The mean *PTG sum score* was 54.63 (SD = 20.24, range: 8–101), the *PTG mean score* was 2.52 (SD = 0.96, range: 0–5). Survivors reported most PTG in *Appreciation of life*, and least in *Spiritual change* (Fig. 1).

**Fig. 2:**
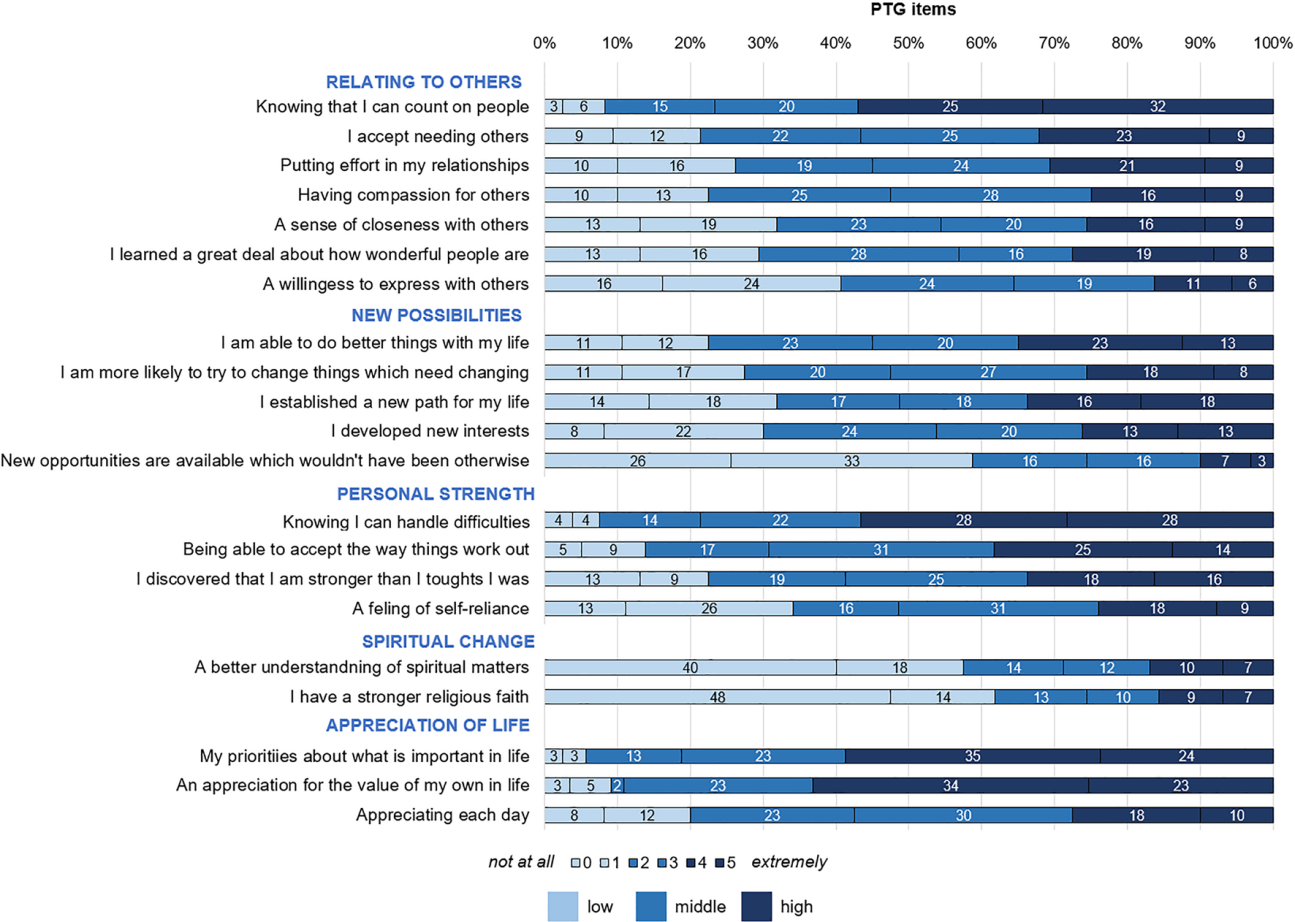
Proportion of participants endorsing the posttraumatic growth (PTG) items measured by the Posttraumatic Growth Inventory (PTGI) at a low (0, 1), middle (2, 3) or high (4, 5) level. Each PTG item is rated using a 6-point Likert scale (0 = *not at all* to 5 = *extremely*)

The four most endorsed items were from *Appreciation of life*: “My priorities about what is important in life”, “An appreciation for the value of my own life”, from *Relating to Others*: “Knowing that I can count on people”, and from *Personal strength*: “Knowing I can handle difficulties” (Fig. 2). The three least endorsed items were from *Spiritual change*: “A better understanding of spiritual matters”, “I have a stronger religious faith”, and from *New possibilities*: “New opportunities are available which wouldn’t have been otherwise”.

### Illness perception

**Fig. 3:**
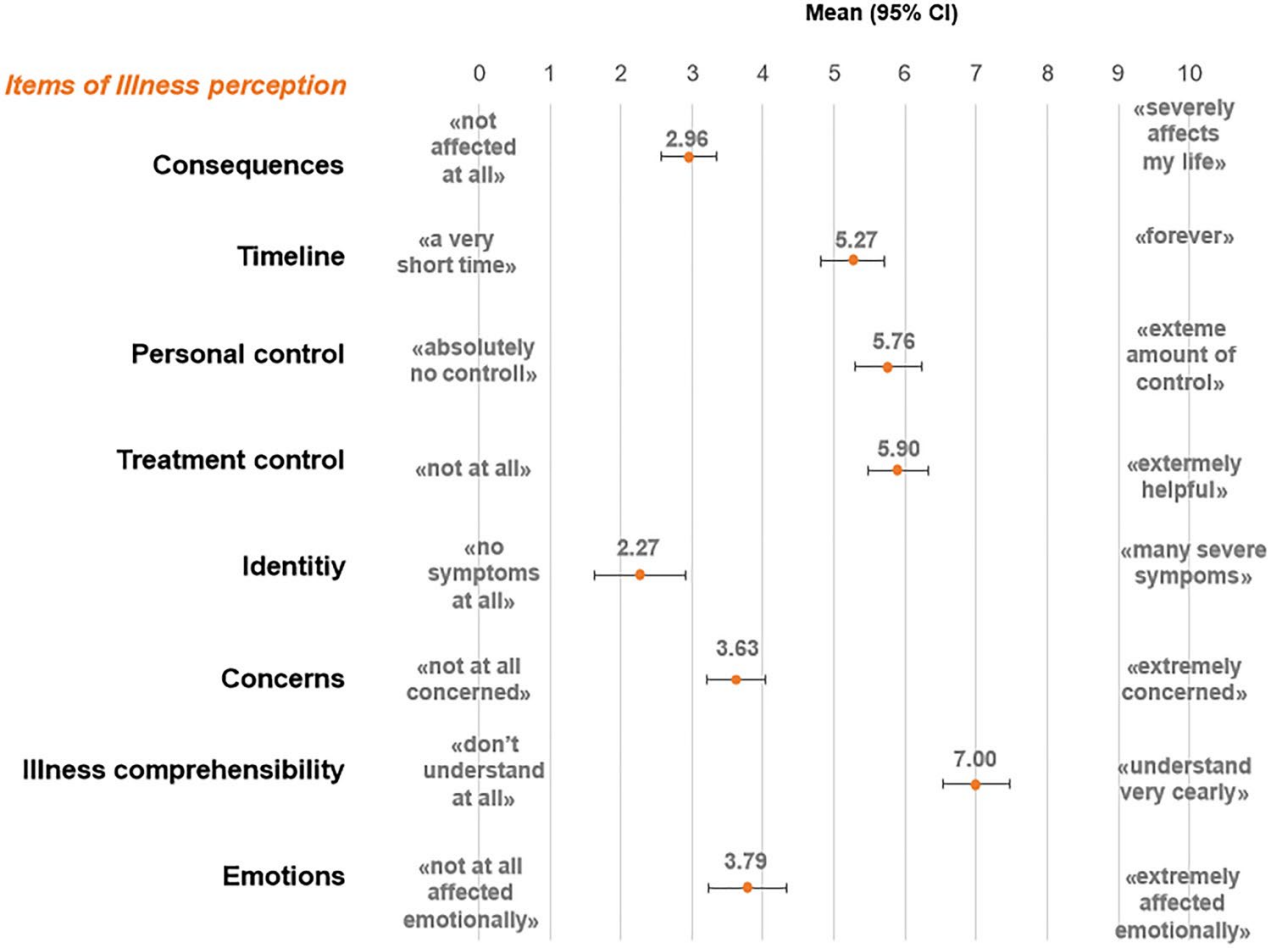
Means and 95% confidence intervals (CI) of the items of illness perception measured by the Brief Illness Perception Questionnaire (BIPQ)

Swiss AYA cancer survivors reported low levels of illness perception on the items *Identity*, and *Consequences* (Fig. 3). The highest mean score was found for *Illness comprehensibility*.

### Associations of PTG with sociodemographic and cancer-related characteristics and illness perception

Neither sociodemographic nor cancer-related characteristics were significantly associated with the *PTG sum score* (Table 2). AYA cancer survivors who perceived follow-up visits as being more helpful (*Treatment control*, p < 0.001) and those with high concerns about the consequences of the illness (*Concerns*, p < 0.001) reported significantly higher levels of PTG.

**Table 2:** Univariable and multivariable linear regressions investigating the associations of sociodemographic and cancer-related characteristics with the PTG sum score

	Univariable linear regression			Multivariable linear regression		
	Coeff	95% CI	p value	Coeff	95% CI	p value
Sociodemographic characteristics						
Sex			0.553			0.331
Male	Ref			Ref		
Female	1.97	− 4.58 to 8.53		3.08	− 3.17 to 9.33	
Age at study			0.448			
20–29 years	Ref					
30–39 years	3.13	− 4.42 to 10.68				
≥ 40 years	− 1.85	− 11.27 to 7.57				
Migration background			0.468			
No	Ref					
Yes	2.84	− 4.88 to 10.56				
Educational achievement			0.164			
Compulsory schooling	− 11.53	− 23.96 to 0.90				
Vocational training	1.63	− 5.60 to 8.86				
Upper secondary education	Ref					
University education	− 5.85	− 14.58 to 6.89				
Employment status			0.744			
Employed	1.88	− 9.47 to 13.22				
Unemployed	Ref					
Partnership			0.455			
Yes	2.87	− 4.70 to 10.44				
No	Ref					
Cancer-related characteristics						
Diagnosis (ICCC-3)			0.918			
Leukemia	Ref					
Lymphoma	− 4.44					
CNS tumor	− 4.12	− 19.52 to 11.27				
Other tumors^a^	− 3.70	− 15.95 to 8.54				
Treatment^b^			0.366			
Surgery	Ref					
Chemotherapy	6.43	− 2.59 to 15.45				
Radiotherapy	3.00	− 1.16 to 2.76				
Age at diagnosis			0.997			
16–21 years	Ref					
> 21–25 years	0.11	− 6.43 to 6.45				
Time since diagnosis			0.802			
5–10 years	Ref					
11–15 years	2.13	− 5.61 to 9.88				
≥ 16 years	− 0.12	− 7.91 to 7.66				
Late effects			0.953			
No	Ref					
Yes	0.21	− 6.98 to 14.70				
Relapse			0.180			
No	Ref					
Yes	5.80	− 3.82 to 20.22				
Illness perception						
Consequences	0.47	− 0.65 to 1.58	0.411			
Timeline	− 0.26	− 1.04 to 0.52	0.507			
Personal control	− 0.34	− 1.41 to 0.73	0.532			
Treatment control	1.80	0.93 to 2.67	** < 0.001**	1.57	0.68 to 2.45	**0.001**
Identitiy	0.51	− 0.77 to 1.78	0.435			
Concerns	2.10	1.02 to 3.18	** < 0.001**	1.67	0.27 to 3.08	**0.020**
Emotions	0.97	− 0.07 to 2.00	0.068	− 0.47	− 1.76 to 0.81	0.467
Illness comprehensibility	− 0.43	− 1.63 to 0.78	0.485			

Bold font indicates statistically significant results

^*^CI confidence interval, *Coeff.* coefficient, *Ref.* reference category

^a^Other tumors: neuroblastoma, renal tumor, hepatic tumor, bone tumor, soft tissue sarcoma, and germ cell tumor

^b^Hierarchically coded: chemotherapy may include surgery, radiotherapy may include surgery and/or chemotherapy

## Discussion

In our explorative study, we found that Swiss AYA cancer survivors, who were on average 12 years from diagnosis, experienced PTG especially in the domains *Appreciation of life, Personal strengths,* and *Relating to others*. Neither sociodemographic nor cancer-related characteristics were significantly associated with levels of PTG. Survivors who thought that follow-up care could help them with the late effects of their illness (*Treatment control*) and survivors who had high concerns about the consequences of the illness *(Concerns*) reported significantly higher levels of PTG.

AYAs with cancer are diagnosed during a unique period of their life, full of psychosocial changes. PTG levels in our sample of Swiss AYA cancer survivors were in a moderate range and comparable to other AYA cancer populations and childhood cancer survivors [4, 5, 37, 38]. This shows that many survivors experience growth after surviving cancer [4, 37, 38]. A qualitative assessment study among survivors of cancer in later adulthood found PTG in the same domains as we did [39].

People who experienced diverse types of traumas (e.g., transportation accidents, sexual assault and abuse, refugee experiences) reported to have some PTG such as a greater appreciation of life [2]. In AYA cancer survivors, this might be due to them having dramatically been made aware of the finite nature of their life. Overcoming major challenges in the past may increase personal strength, pride in accomplishments and self-confidence, and thus the ability to overcome future struggles. However, the age between 20 und 40 years is usually characterized by psychological maturation, e.g., people becoming more confident [40]. This corresponds to the average age of our sample, and it is thus unclear if the strengthened personality is due to the cancer experience or rather due to normal maturation.

Our population reported to have closer and more meaningful relationships with other people. Their cancer seems to have made them aware of how much they can rely on those around them. Seeking social support is also important for adult cancer survivors to experience psychological growth [4]. Again, it should be noted that young adulthood is characterized by more intense friendships, increasingly serious partnerships, and for some by starting a family. A large proportion of our sample indicated that they were in a partnership, which could be a contributing factor in explaining the intensified relationships with others.

*Spiritual change* was not commonly reported by survivors in our sample similar to a study among survivors of childhood cancer in Switzerland [5]. This is in accordance with the declining importance of religion in Switzerland. Since the second half of the twentieth century, membership in most traditional faith communities has steadily declined in Switzerland, and people tend to ascribe less importance to religion [41].

In terms of illness perception, participants reported experiencing few symptoms of their past illness, understanding the consequences of their illness, and not being greatly affected by the consequences of their illness at time of study. These three results indicate the overall positive illness perceptions in our sample. However, our sample was quite young and on average 12 years after diagnosis. More severe and life-threatening chronic health conditions may develop later in their life [42]. Studies following these survivors into middle and older adulthood might reveal a less positive picture.

Regarding illness perception, two items were associated with higher levels of PTG. First, AYA cancer survivors who indicated that follow-up care can help them with the late effects of their illness (*Treatment control*) reported significantly higher levels of PTG. Positive attitudes towards follow-up care can increase intended and actual attendance of follow-up care in this population [31]. Second, high concerns about the consequences of the illness (*Concerns*) were significantly associated with higher levels of PTG as well. The development of PTG requires the struggle with a traumatic event. In the aftermath of this event, fundamental beliefs about the world need to be reformulated and re-examined [2, 43]. In this process, growth and distress can occur simultaneously, and both positive and negative aspects of the event remain in the experience of people who report PTG [3]. Survivors, although concerned about the consequences of their past illness, may positively reframe the traumatic event and find meaning in it. That two opposing constructs may be related has also been indicated by a recent meta-analysis showing that PTG and stress (posttraumatic stress, posttraumatic disorder, distress) were positively associated in cancer patients and survivors [44].

In our sample, neither sociodemographic nor cancer-related characteristics were significantly associated with PTG. There is mixed evidence regarding this in the literature. Some studies found no clear associations of sociodemographic characteristics and PTG [4, 45, 46]. Sex and/ or age at diagnosis were the most frequent exception, with female and younger survivors reporting higher PTG [10, 38, 47, 48]. Furthermore, the few cancer-related characteristics that were positively associated with higher levels of PTG in other studies with cancer survivors were chemotherapy, and shorter time since treatment [38, 47]. Generally, it seems that specific cancer-related characteristics are not crucial for the development of PTG.

### Study limitations and strengths

A limitation of this study is its cross-sectional nature not allowing causal conclusions. Moreover, the BIPQ was originally developed to capture illness perceptions during acute illness rather than survival. Therefore, interpretation and comparison with other studies should be made with caution. Our sample came from one German-speaking region of Switzerland and generalizations to other countries might be limited. Due to the limited number of participants, the results of the multivariable linear regression analyses should be interpreted in an exploratory manner. The response rate was relatively low (41.1%), however it is comparable to other studies in the field [49]. Another limitation of our study is that we used a less broad age range (15–25 years) than is commonly assumed as AYA age range in the literature (15–39 years). Therefore, our results may only apply to AYA being diagnosed at a relatively younger age. A strength of our study is the population-based sampling of AYA cancer survivors. The participants of our study were comparable to non-participants suggesting that the study sample is representative for AYA cancer survivors in a large and diverse region of Switzerland [28]. Furthermore, we had cancer-related information available from the Cancer Registry of Zürich and Zug. By using the widely known PTGI, a comparison with other studies was possible.

### Implications

There is growing evidence that young survivors of cancer experience positive outcomes such as PTG. Knowing and acknowledging this possible positive development may help survivors to grow and to reintegrate into daily life after cancer. Despite suffering and getting through the disease, knowing that cancer can be associated with PTG in the long term can be a potential source of hope for patients and survivors and those close to them. However, it should be kept in mind that negative consequences do not disappear despite PTG and that not all survivors experience positive outcomes. It is important to emphasize that the presence of PTG does not negate ongoing distress. Care should be taken to ensure that survivors do not feel bad about not experiencing PTG or even pressured to report positive experiences even if they are not part of their lived reality. Conversations about PTG might help to actively promote survivors’ well-being and make them aware of potential positive outcomes after cancer. Knowing how illness perception is associated with PTG may additionally help to understand how to influence the development of PTG. So far, interventions have focused on other patient and survivor groups, and more research is needed to learn more about particularities in survivors of AYA cancer. Our study adds to the existing body of literature by investigating both PTG and disease perception in a population of survivors of AYA cancer.

## Conclusions

In our study, all survivors of AYA cancer reported at least some PTG, mostly greater *Appreciation of life*, a sense of *Personal strength*, and intensified *Personal relationships*. Higher levels of PTG were reported by survivors who thought that follow-up care could help them with late effects of their illness and who were more concerned about the consequences of cancer. Knowing how illness perception is related to PTG may help to raise awareness and to promote PTG in survivors of AYA cancer.

## Data Availability

The data that support the findings of this study are available from the corresponding author upon reasonable request.

## Abbreviations

AYA: Adolescent and young adult
BIPQ: Brief illness perception questionnaire
CI: Confidence interval
CNS: Central nervous system
ICCC-3: International classification of childhood cancer-third edition
n: Number
n.a.: Not available
PTG: Posttraumatic growth
PTGI: Posttraumatic growth inventory
Ref.: Reference category
SD: Standard deviation
